# Five Hours Total Sleep Deprivation Does Not Affect CA1 Dendritic Length or Spine Density

**DOI:** 10.3389/fnsyn.2022.854160

**Published:** 2022-03-14

**Authors:** Alvin T. S. Brodin, Sarolta Gabulya, Katrin Wellfelt, Tobias E. Karlsson

**Affiliations:** ^1^Department of Neuroscience, Karolinska Institutet, Solna, Sweden; ^2^Institute of Neuroinformatics, University of Zurich and ETH, Zürich, Switzerland

**Keywords:** sleep deprivation, plasticity, spines, dendrite, hippocampus

## Abstract

Sleep is essential for long term memory function. However, the neuroanatomical consequences of sleep loss are disputed. Sleep deprivation has been reported to cause both decreases and increases of dendritic spine density. Here we use Thy1-GFP expressing transgenic mice to investigate the effects of acute sleep deprivation on the dendritic architecture of hippocampal CA1 pyramidal neurons. We found that 5 h of sleep deprivation had no effect on either dendritic length or dendritic spine density. Our work suggests that no major neuroanatomical changes result from a single episode of sleep deprivation.

## Introduction

Sleep disturbance is a feature of several prevalent brain diseases, including Alzheimer’s disease, anxiety, depression and schizophrenia (Wulff et al., 2010), and even our distant ancestors were plagued by poor sleep (Ancoli-Israel, 2001). There is widespread concern that our modern lifestyle is making sleep disorders more prevalent. Lifestyle factors such as altered working patterns and use of electronic devices have been proposed to fuel an epidemic of sleep deprivation (Shochat, 2012). However, whether modern humans truly sleep less is debated (Yetish et al., 2015).

Adequate sleep is essential to cognitive function across the animal kingdom (Keene and Duboue, 2018), and typically consists of both rapid eye movement sleep (REM-sleep) and slow-wave sleep (SWS; Cousins and Fernández, 2019). The homeostatic necessity of SWS seems to be stronger than that of REM-sleep, as the proportion of SWS increases after sleep deprivation (Dijk, 2009; Rodriguez et al., 2016). In healthy humans, the first half of a night’s sleep is dominated by SWS with increasing proportions of REM-sleep observed during the latter half.

Particularly strong, and particularly studied, is the association between sleep and memory function (Cousins and Fernández, 2019). Sleep prior to learning is necessary for proper encoding. Extended periods of wakefulness impair attention (Lim and Dinges, 2010; Krause et al., 2017), and induction of long-term potentiation is impaired after sleep deprivation (Campbell et al., 2002). Encoding seems to be reliant on SWS, as selectively disturbing SWS disrupts encoding the following day (Van Der Werf et al., 2009). The role of REM-sleep preceding learning is less clear, with one study reporting that disturbing REM has no effect on subsequent memory encoding (Kaida et al., 2015). Sleep following learning is also essential, as learning events followed by sleep are retained better than those followed by a period of wake (Igloi et al., 2015).

The mechanism behind the necessity and behavioural effects of sleep are not fully understood. One influential theory for the functional role of sleep is the synaptic homeostasis hypothesis (SHY). SHY states that experiences during the day are encoded by increases in synaptic weight, necessitating homeostatic downscaling during sleep to prevent overexcitability (Tononi and Cirelli, 2014). Reduced memory function would result from impaired consolidation when sleep is disturbed, as well as from prolonged wakefulness causing general overexcitability that impairs signal recognition and hinders further potentiation (Tononi and Cirelli, 2014).

A key prediction by SHY is that global synaptic strength should increase during periods of wake and decrease during sleep, as opposed to homeostatic downscaling occurring continuously. This prediction has been tested in several ways. Sleep deprivation has been found to lower seizure thresholds in both humans (Malow, 2004) and animal models (Giorgi et al., 2014). However, decreased firing thresholds have only been demonstrated in epileptic patients, not healthy subjects (Giorgi et al., 2014). At the structural level, *in vivo* two-photon imaging has demonstrated an average decrease of spine size and GluA1 content during sleep, with a small subset of spines instead growing and increasing their GluA1 content (Diering et al., 2017). Phosphorylation of several plasticity-related proteins also vary in a manner suggesting synaptic potentiation during wake and downscaling during sleep (Vyazovskiy et al., 2008).

Histological studies of hippocampus offer conflicting evidence with regards to how sleep deprivation affects synapse numbers and dendritic structure. Some studies show that sleep deprivation increases spine density in CA1 as predicted by SHY (Gisabella et al., 2020). However, other studies show that sleep deprivation causes a loss of spines in CA1 (Havekes et al., 2016) and the dentate gyrus (Raven et al., 2019), even shortening the dendritic tree as a whole in CA1 (Havekes et al., 2016). Should sleep deprivation cause the dendritic arbour to shrink and reduce synapse numbers, rather than the opposite, this would be a strong argument against SHY and necessitate looking for alternate explanations regarding the function of sleep (Raven et al., 2018). In this study we utilise recent innovations of tissue imaging to determine the effects of sleep deprivation on dendritic arbour and spine density in the CA1 of hippocampus.

## Materials and Methods

### Animals

Male Thy1-GFP line M mice obtained from Jackson (Jackson Laboratory, Bar Harbor, ME, United States) aged 12 to 44 weeks were used for all experiments. Mice were housed in a 12 h light cycle with lights on at 7 AM and food and water *ad libitum*. Animals were split into the three groups based on cages in a pseudorandom schedule.

Detailed data on the animals are available in Supplementary Table 1.

### Ethics Declaration

All animal experiments were approved by the Northern Stockholm Animal Ethical Committee. All experiments were performed in accordance with Swedish laws and regulations on animal experimentation. The study is reported in accordance with ARRIVE guidelines.

### Blinding

Investigators were not blinded while performing sleep deprivation as this was not deemed possible. Investigators were not aware of group allocation during tissue processing, imaging or image analysis.

### Sleep Deprivation

Animals were kept in their home cages with 4–6 animals per cage for the duration of the experiments. Mice were handled daily for 3 days prior to the experiment. They were sleep deprived from 7 AM to 12 AM using the gentle handling method (Colavito et al., 2013). When mice were about to fall asleep they were awoken by, in ascending order; gently tapping the cage, gently tilting the cage, or disturbing the bedding. In order to minimise the stress caused by the intervention, mice were kept in their home cages during the experiment and were sleep deprived together with their cagemates. To ensure adequate sleep deprivation, mice were continuously monitored by two experimenters who administered the sleep deprivation together. Enrichment material and housing was removed so that mice could not hide from view. Control mice, and mice undergoing recovery sleep, were kept in the same room as the sleep deprived mice. A total of 10 mice underwent sleep deprivation, 8 mice underwent sleep deprivation followed by recovery sleep and 12 control mice slept undisturbed. No animals were excluded from further analysis, no exclusion criteria were set.

### Tissue Processing

At the end of the experiment mice were anesthetised with pentobarbital and perfused with 4% formalin in phosphate buffered saline (PBS). Whole brains were removed and post-fixed in 4% formalin for 24 h, and subsequently stored in PBS. One hemisphere was used for light sheet imaging for dendrite tracing and one hemisphere was used for confocal imaging for spine analysis. Processing for confocal imaging was performed after processing for light sheet imaging, and several hemispheres were lost in the intervening time which caused the n to be lower for spine analysis.

Brains were embedded in an agarose gel and sectioned by vibratome into 300 μm coronal sections for dendrite tracing and 200 μm coronal sections for spine analysis.

The sections were cleared using an adapted SeeDB2 protocol (Ke et al., 2016). Briefly, brain tissue was incubated in serial solutions of 2% Saponin (Teknolab, Kungsbacka, Sweden) with increasing concentrations of Omnipaque 350 (Apoteket, Stockholm, Sweden).

### Imaging

For dendrite tracing, images were acquired using a Zeiss Lightsheet Z.1 microscope (Light Sheet Microscopy Pilot Facility, KTH), equipped with a 20x/1.0 NA objective and excitation was performed by two LSFM 10x/0.2NA illumination objectives.

For spine analysis, tissue was stained using an anti-GFP alexa 555 antibody to enhance signal. Images were acquired with an LSM 800 airy microscope (Carl Zeiss, Oberkochen, Germany, provided by Biomediucm Imaging Core facility), with a 63x oil (NA 1.4). The microscope was set to highest resolution. Instead of using a standard pinhole an array detector is used resulting in higher resolution. Z stacks of apical dendrites were obtained with a pixel resolution of 0.04 μm in XY and a 0.2 μm interval between Z slices.

### Dendritic Tree Analysis

The lightsheet image stacks were stitched together using manual alignment in Arivis Vision4D. Apical dendrites of the CA1 pyramidal neurons were traced by a blinded investigator using the Autopath semiautomated tracing of Imaris version 9.6.0 (Filament tracker licence, Imaris).

#### Spine Analysis

An investigator blinded to the treatment of the mice acquired all images. Sections were selected from the dorsal hippocampal neurons and one 1–2 sections per mouse thickness 160 μm was analysed until 10 neurons where reached (if possible). Neurons were considered for inclusion if they were fully labelled with flurophore without apparent defects or focalities, exhibited a pyramidal morphology of the cell body and was located in CA1. For each neuron the apical dendrite was followed until a suitable 3–5 order dendrite was found. Suitable was defined as an isolated dendrite with straight course predominantly in the x-y plane. We chose dendrite 3–5 based on the findings of Havekes et al. (2016). The dendrites were all located around 100 μm–200 μm from the soma within the stratum radiatum. Dendrites analyzed per animals is available in Supplementary Table 1.

### Statistical Analysis

Statistical analysis was performed using R, version 3.6.3, with RStudio, version 1.2.5033.

A mixed effects linear model was constructed using the R package lme4 version 1.1-23, with the specified dendritic parameter given as a function of treatment, with mouse identity as a random effects factor. This test was used to account for the nesting of data when analysing several neurons per mouse. The dendritic length and spine density was assessed to be normally distributed using a normal Q-Q plot. Significance testing of treatment effects was performed using the R package lmerTest v. 3.1-3. A *p*-value of less than 0.05 was considered significant. Unless otherwise noted data are presented as means with error bars denoting 95% confidence intervals.

## Results

### Sleep Deprivation Does Not Alter the Shape of the Dendritic Tree

**FIGURE 1:**
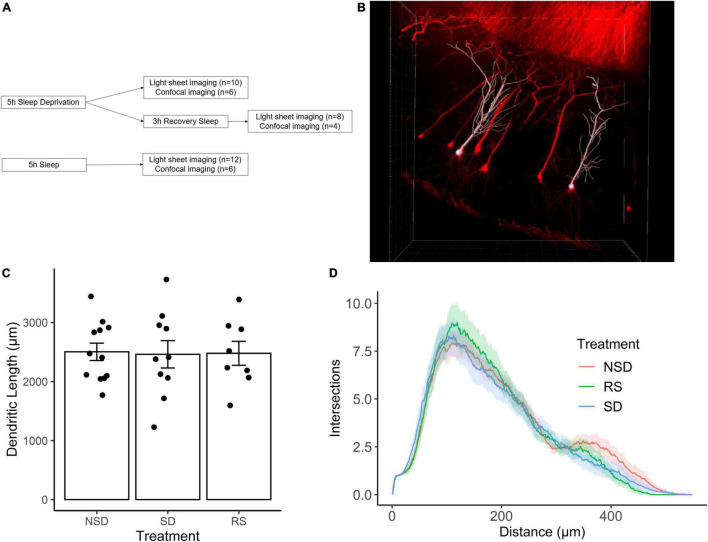
Sleep deprivation does not impact dendritic length in CA1 neurons of the hippocampus. **(A)** Experimental outline. **(B)** Representative image of Thy1-GFP CA1 neurons (red) and semi-automated tracing using IMARIS (white). **(C)** Shows the average length of apical dendrites per treatment: 5 h sleep deprivation (SD, *n* = 10 mice), SD followed by 3 h recovery sleep (RS, *n* = 8 mice) or no sleep deprivation (NSD, *n* = 12 mice). Error bars represent SEM. **(D)** Averaged sholl diagram of dendrites per treatment, the shaded area denotes the 95% confidence interval.

We first set out to investigate the effects of sleep deprivation on dendritic length. Thy1-GFP mice were subjected to one of the following treatments: sleep deprivation for 5 h (*n* = 10 mice), no sleep deprivation (*n* = 12 mice), or 5 h of sleep deprivation followed by 3 h of recovery sleep (*n* = 8 mice) (Figure 1A). Using light-sheet imaging and semi-automated tracing, apical dendrites from 7–8 CA1 pyramidal neurons were traced from each animal (Figure 1B).

We found no significant effect on overall dendritic length by sleep deprivation [−2%, 95% CI (−23%, + 19%), *p* = 0.847], or recovery sleep [−1%, 95% CI (−24%, + 21%), *p* = 0.923] (Figure 1C). It is possible that sleep deprivation does not significantly alter the overall length of the dendritic tree, but instead affects a particular part of the dendritic tree. To investigate this possibility, we performed Sholl analysis of the traced neurons, and found that the shapes of the Sholl diagrams did not differ significantly between treatments (Figure 1D).

### Sleep Deprivation Does Not Have Major Effects on Dendritic Spine Density

**FIGURE 2:**
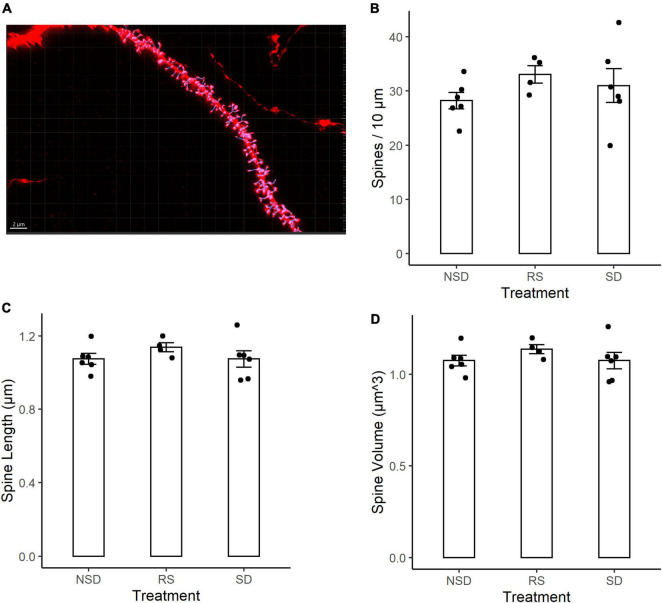
No detectable differences in spine density or morphology in CA1 neurons after sleep deprivation. **(A)** Representative picture of Thy1-GFP third-order dendritic branch used for spine counting. Scale bar 5 μm. **(B)** 5 h sleep deprivation (SD, *n* = 6 mice) or SD followed by 3 h recovery sleep (RS, *n* = 4 mice) does not impact dendritic spine density as compared to no sleep deprivation (NSD, *n* = 6 mice). **(C)** Shows the average length of the measured spines. **(D)** Shows the average volume of the spines. All values are mean ± SEM.

We next analyzed changes in the dendritic spines of CA1 neurons. Dendritic spines have been shown to be more motile structures than dendritic branches, and thus possibly more susceptible to sleep deprivation. Using tissue from the same mice as for the dendritic length analysis, tissue was imaged using an LSM-800 airyscan confocal microscope. Dendritic spines were counted on 3rd to 5th order apical dendrites of CA1 pyramidal neurons from sleep deprived (*n* = 6 mice), recovery sleep (*n* = 4 mice) or non-sleep deprived (*n* = 6) mice (Figure 2A). Spines were counted on an average of 6.3 dendrites per mouse.

Representative image of a dendrite with IMARIS reconstruction (Figure 2A). There were no significant effects of sleep deprivation on dendritic spine density (Figure 2B). Compared to non-sleep deprived mice there was a non-significant higher mean spine density in sleep deprived mice [+ 9%, 95% CI (−13%, + 31%), *p* = 0.464] and recovery sleep mice [+ 17%, 95% CI (−8%, + 42%), *p* = 0.211] (Figure 2B). The shape of dendritic spines were unchanged, with no significant effects by any treatment on the average length of dendritic spines (Figure 2C) or the volume of dendritic spines (Figure 2D). There was no difference in dendrite diameter across the groups (Supplementary Figure 1A). No major differences were detected in branch order between groups (Supplementary Figure 1B), and no large variations in spine density occurred as a function of branch order (Supplementary Figure 1C).

## Discussion

That adequate sleep is vital for the formation of lasting memories has been clearly established (Cousins and Fernández, 2019), but the mechanisms involved remain disputed. Charting the neuroanatomical changes caused by sleep deprivation promises to offer clues to its function, but conflicting evidence points to both synapse loss (Havekes et al., 2016; Raven et al., 2019) and synapse gain during sleep deprivation (Gisabella et al., 2020). Dramatically, it has even been reported that brief sleep deprivation can shorten the dendritic tree as a whole in CA1 pyramidal cells (Havekes et al., 2016).

In this study we found no major changes in the dendritic tree of CA1 neurons after brief (5 h) sleep deprivation. This was true for both overall dendritic length and for the shape of the dendritic arbour as shown by Sholl analysis. There are several methodological differences that could explain the discrepancy between our results and those of Havekes et al., where a substantial (∼30%) reduction of dendritic length after sleep deprivation was found. Where they used Golgi staining, we instead used transgenic Thy1-GFP mice. The average apical dendrite of our control group was traced to 2500 μm, while the control neurons’ apical dendrites in Havekes et al., were 1200 μm. This could indicate that our GFP-labelled neurons were more completely traced, which could be one reason for the discrepant results.

Differences in statistical methods could also lead to discrepancies in results. A common practise in neuroanatomical studies is to treat studied neurons as one homogenous group with regards to statistical analysis. However, disregarding the inter-relatedness of neurons from the same animal risks underestimating the variance in the sample and leading to too small sample sizes being used (Wilson et al., 2017). A mixed effects model avoids this problem, but the statistical tests used specifically for the neuroanatomical analysis have unfortunately not been listed in previous studies on the topic. To our knowledge, ours is the best powered study to date on the effect of sleep deprivation on dendritic length. Nevertheless, the modest size of our study limits its precision; both a modest but physiologically significant increase or a similar decrease in dendritic length after sleep deprivation are compatible with our data [95% CI (−23%, + 19%)].

Studies using *in vivo* transcranial imaging support the view that while spines are highly motile, the dendritic tree as a whole is largely static. Individual dendritic branches have been imaged successfully over several weeks (Holtmaat and Svoboda, 2009), a feat which would be impossible if large-scale remodelling of the dendritic arbour was commonplace. However, these experiments did not feature sleep deprivation, and cannot rule out dendritic changes occurring specifically in this setting.

Our findings on dendritic spines speak against sleep deprivation causing a loss of dendritic spines. No significant changes in spine number or morphology were found, but a non-significant increases of spine density after sleep deprivation was noted. Although the power of this analysis was hampered by the loss of tissue from several mice, precluding certainty regarding the presence of an increase in spine density, major decreases in spine density do not seem compatible with our data.

The dynamics of dendritic spines during sleep have been more extensively studied than those of the dendritic tree as a whole. Several studies have found that spines are pruned during sleep (Vyazovskiy et al., 2008; de Vivo et al., 2017; Diering et al., 2017; Li et al., 2017,?). This has been found to occur during both SWS (Feld and Born, 2017) and REM sleep (Li et al., 2017). Newly formed spines are selectively maintained during this process, resulting in a net loss of spines while consolidation of strong synapses occurs (Li et al., 2017). *In vivo* imaging thus provides support for the idea that the net effect of sleep is a pruning of synaptic spines, which agrees with some histological studies (Spano et al., 2019; Gisabella et al., 2020) but not others (Havekes et al., 2016; Raven et al., 2019).

Notably, sleep deprivation entails not only the absence of sleep, but also an abnormally long period of wake, which may have its own effects. In addition, all sleep deprivation methods are associated with some level of stress (Nollet et al., 2020). Gentle handling was chosen as the method of sleep deprivation in the present study as it is less stressful than many other methods (Nollet et al., 2020), but not stress-free. The method is hard to standardise between experimenters, and inter-experimenter differences in odour, training and demeanour could all affect the stress levels of the sleep deprived animals. Indeed, stress has been shown to adversely affect dendritic spine density (Leuner and Shors, 2013). In contrast to many other studies, the mice in our study were not singly housed. This was done to minimise the stress caused by the experiment, with efficacy of sleep deprivation safeguarded by having multiple experimenters administer the sleep deprivation. Based on experimenter observations sleep deprivation was total, but differences in stress level and efficacy of sleep deprivation must be considered as potential causes of the divergent results. Besides this difference no substantial deviations from the gentle handling protocol of previous, similar studies were made (Havekes et al., 2016; Gisabella et al., 2020). Our study shifts the balance of evidence away from pruning of dendritic spines during sleep deprivation, and to a lesser extent away from sleep deprived induced shortening of dendrites. This is an important issue with implications for our understanding of the role of sleep as a whole, but before final conclusions can be drawn there is a need for further, statistically robust studies on this topic.

## Data Availability Statement

The raw data supporting the conclusions of this article will be made available by the authors, without undue reservation.

## Ethics Statement

The animal study was reviewed and approved by the Northern Stockholm Animal Ethical Committee.

## Author Contributions

AB, SG, and KW performed animal experiments and tissue preparation. AB and SG carried out tissue imaging. AB, SG, and TK analysed the data. AB and TK conceived of the study and prepared the manuscript. All authors contributed to the article and approved the submitted version.

## Conflict of Interest

The authors declare that the research was conducted in the absence of any commercial or financial relationships that could be construed as a potential conflict of interest.

## Publisher’s Note

All claims expressed in this article are solely those of the authors and do not necessarily represent those of their affiliated organizations, or those of the publisher, the editors and the reviewers. Any product that may be evaluated in this article, or claim that may be made by its manufacturer, is not guaranteed or endorsed by the publisher.
