# Supplementary material for: Five Hours Total Sleep Deprivation Does Not Affect CA1 Dendritic Length or Spine Density
Source: Front Synaptic Neurosci. 2022 Mar 14;14:854160. doi: 10.3389/fnsyn.2022.854160 (PMC8964138; doi:10.3389/fnsyn.2022.854160)
Supplement: Supplementary file 1 [file Table_1.pdf]

Mouse ID	Treatment	Age (in weeks)	Neurons Dendritic analysis	Average Dendrite Length	Dendrites for spine analysis	Total spines	Average dendrite length for spine analysis	Spines per 10 µm	Order (average)	Length 3rd order	Length 4:th order	Length 5:th order
K615_5	NSD	25	7	1 772 µm								
K615_6	NSD	25	7	2 056 µm								
K615_7	NSD	15	7	2 474 µm								
K615_8	NSD	15	8	2 407 µm								
K616_5	NSD	25	8	2 099 µm								
K616_7	NSD	9	8	3 442 µm	5	509	36 µm	28,8	4,0	78,5	21,6	68,3
K616_8	NSD	18	8	2 872 µm								
K616_9	NSD	18	7	2 044 µm	5	590	42 µm	28,8	4,0	100,1	53,3	54,5
K631_10	NSD	27	8	2 913 µm	7	776	36 µm	30,3	4,3	36,8	36,8	108,1
K631_5	NSD	25	8	2 835 µm	8	874	46 µm	22,6	4,1	37,6	30,2	106,4
K631_7	NSD	25	8	3 013 µm	7	928	39 µm	33,5	3,7	181,8	32,1	57,1
K631_9	NSD	25	8	2 117 µm	7	641	35 µm	26,8	3,7	91,8	31,0	32,6
**NSD average**		**21,0**	**7,7**	**2 504 µm**	**6,5**	**719,7**	**39 µm**	**28,5**	**4,0**	**87,8**	**34,2**	**71,2**
K615_10	RS	44	8	1 596 µm								
K615_11	RS	25	7	2 190 µm								
K615_9	RS	44	8	2 067 µm								
K616_11	RS	14	7	2 238 µm	6	519	28 µm	31,5	3,5	95,9	29,1	0,0
K616_12	RS	14	8	3 389 µm	8	1146	39 µm	35,2	4,1	79,4	51,1	150,9
K631_13	RS	19	8	2 946 µm								
K631_18	RS	19	8	2 887 µm	6	1018	44 µm	36,2	4,3	39,2	32,2	153,6
K631_20	RS	19	8	2 516 µm	5	393	27 µm	29,3	3,8	22,4	40,5	0,0
**RS average**		**24,5**	**7,8**	**2 479 µm**	**6,3**	**769,0**	**34 µm**	**33,1**	**3,9**	**79,5**	**35,0**	**71,5**
K615_2	SD	25	7	2 955 µm								
K615_3	SD	43	7	1 226 µm								
K615_4	SD	20	6	3 728 µm								
K616_1	SD	18	7	2 380 µm	8	1077	32 µm	42,7	3,9	99,5	23,4	65,8
K616_2	SD	18	8	2 414 µm	5	438	31 µm	28,1	3,6	59,6	24,5	0,0
K616_3	SD	19	8	2 060 µm	2	253	34 µm	35,5	3,5	26,4	0,0	0,0
K616_4	SD	19	8	1 717 µm	6	351	27 µm	19,9	3,3	110,1	27,3	0,0
K631_1	SD	19	8	2 125 µm								
K631_2	SD	31	8	3 113 µm	7	790	39 µm	29,0	4,3	71,5	25,9	159,8
K631_3	SD	31	8	2 898 µm	8	747	31 µm	30,8	4,0	54,9	18,0	101,2
**SD average**		**23,9**	**7,5**	**2 462 µm**	**6,0**	**609,3**	**32 µm**	**31,0**	**3,8**	**65,5**	**24,3**	**49,8**
