# Supplementary figures and images for: Five Hours Total Sleep Deprivation Does Not Affect CA1 Dendritic Length or Spine Density
Source: Front Synaptic Neurosci. 2022 Mar 14;14:854160. doi: 10.3389/fnsyn.2022.854160 (PMC8964138; doi:10.3389/fnsyn.2022.854160)

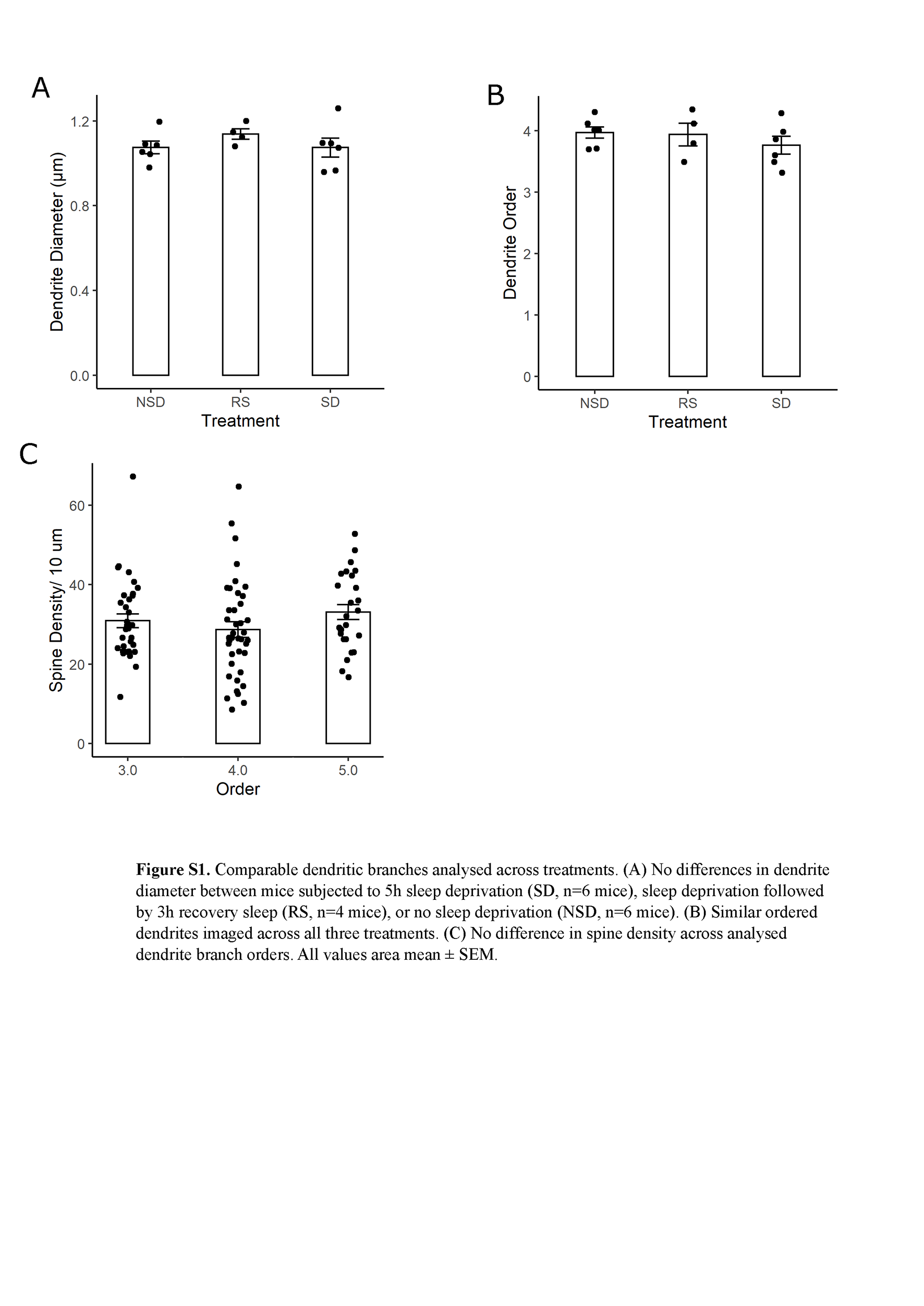

Supplement: Supplementary file 2 [file Image_1.TIF]
